# Outcomes and patient perspectives of a novel virtual spinal referral pathway in a non-specialist centre

**DOI:** 10.1007/s11845-024-03742-1

**Published:** 2024-06-28

**Authors:** Lyndon Yerng Hsien Low, Kevin Clesham, Susan E.Murphy, Ruari MacNiocaill, Marcus Timlin, May Cleary

**Affiliations:** 1 Department of Trauma & Orthopaedics, University Hospital Waterford, Dunmore Road, Waterford, Ireland; 2 National Orthopaedic Spine Centre, Mater Misericordiae University Hospital, Eccles Street, Dublin 7, Ireland; 3 University College Cork, College Road, Cork, Ireland

**Keywords:** Orthopedics, Patient satisfaction, Referral and consultation, Spinal siseases, Telemedicine

## Abstract

**Introduction:**

In Irish orthopaedic centres without dedicated spinal services, the care of patients is facilitated through tertiary referral centres in Dublin, Cork & Galway. The outpatient waiting list for elective spinal opinion remains lengthy and challenging. Previous practice in University Hospital Waterford (UHW) necessitated an assessment with a local non-spinal orthopaedic specialist following a GP referral, incurring up to a 2-year wait prior to subspecialist spinal referral. These patients subsequently incurred a further wait for an appointment at the tertiary referral centre. A novel virtual spine clinic in collaboration with the Mater Misericordiae University Hospital (MMUH) was developed to fast-track this process.

**Aims and methods:**

A retrospective study was performed to audit efficiency by assessing time to initial consultation and time to virtual consultation, treatment outcomes, and patient satisfaction using an adapted patient-satisfaction questionnaire (PSQ-18) and a semi-structured interview. This study reflected the unique nature of patient experience in this pathway.

**Results:**

The median time from referral to being seen in an in-person rapid access physiotherapist combined orthopaedic clinic was 185 days. The median time from initial consultation to virtual consultation was 36 days. The median time interval from virtual consultation to intervention was 110 days. Twenty percent of patients underwent surgery, 14% were further seen in the MMUH outpatients, 7% managed with the trial of physiotherapy, 7% required no follow-up, and 50% planned for radiologically guided spinal injections.

**Discussion and conclusion:**

This novel pathway is efficient for orthopaedic units without a dedicated spinal service. This can easily be replicated across other orthopaedic centres with minimal cost implications.

## Introduction

In the absence of dedicated spinal services in Irish regional centres, the care of patients is facilitated through tertiary referral centres in Dublin, Cork & Galway. Although emergency spinal conditions have a well-established, efficient referral pathway to tertiary centres, non-emergent or elective spinal opinion remains lengthy and challenging. Following GP referral, previous practice in University Hospital Waterford necessitated an assessment with a local non-spinal orthopaedic specialist, incurring up to a 2-year wait before a subspecialist spinal referral for an opinion. These patients subsequently incurred a further wait for an appointment at the tertiary referral centre. A novel virtual spine clinic in collaboration with the Mater Misericordiae University Hospital (MMUH) was developed to fast-track this process.

## Aims and methods

In collaboration with the National Orthopaedic Spine Centre at the Mater Misericordiae University Hospital (MMUH) Dublin, a novel virtual (video) orthopaedic spine clinic has been developed for potential spinal orthopaedic surgical candidates from UHW. This quality improvement initiative was established in September 2021. Patients are screened in advance at the in-person Orthopaedic/combined musculoskeletal (MSK) clinic in UHW, and if their clinical presentation and investigations are indicative of pathology that may warrant surgical intervention, they are listed for the Virtual (video) Spine clinic opinion.

**Fig. 1:**
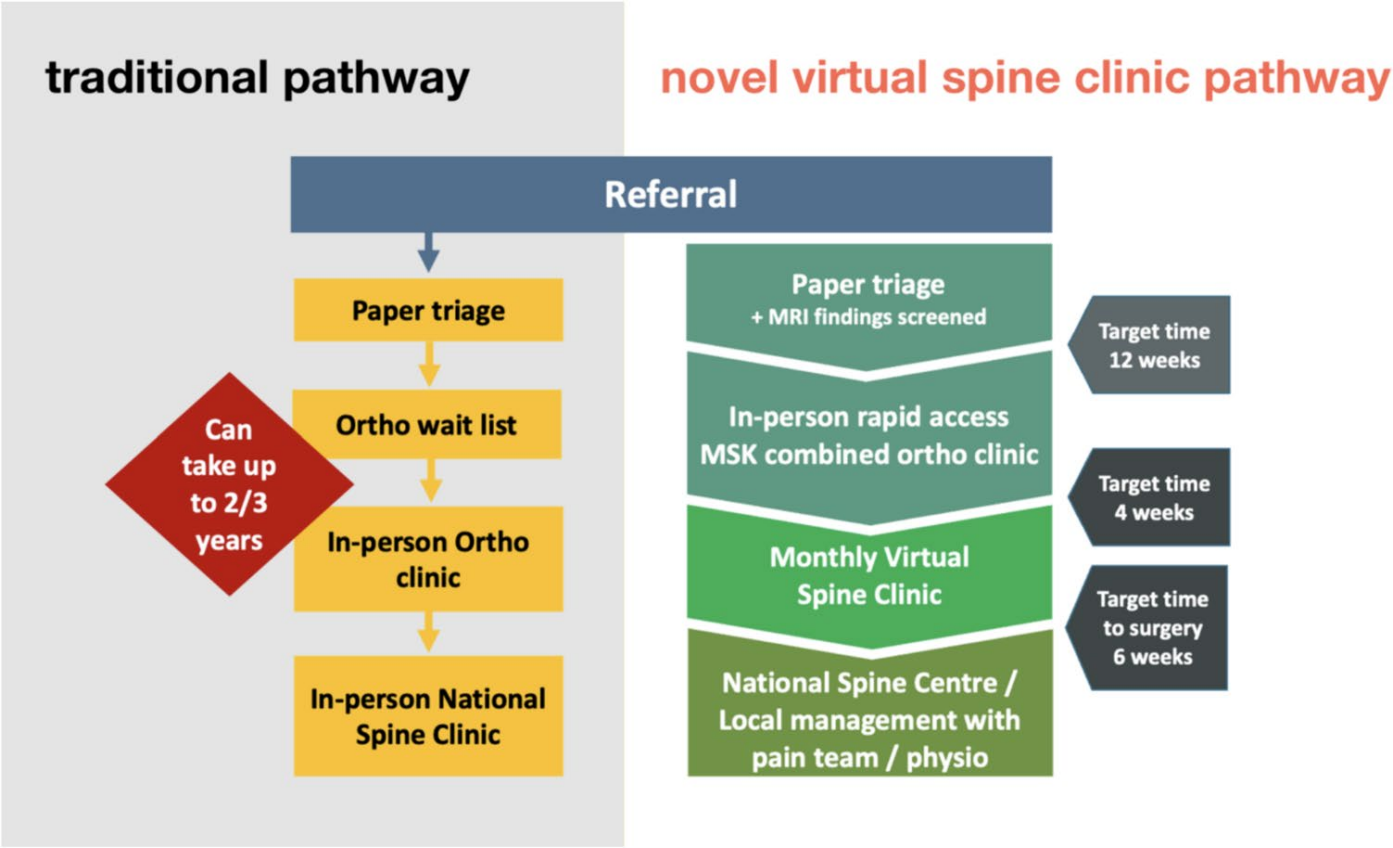
Outline of the traditional and novel virtual spinal referral pathway

A Rapid Access Spinal pathway was developed to enable the new virtual pathway to function to its greatest efficiency. Traditionally, all spine referrals were managed chronologically with non-urgent elective conditions. The ratio of orthopaedic consultant surgeons per population is well over double the recommended European and UK national average of 1 per 25,000 people [1, 2]. Consequently, the UHW Orthopaedic waiting lists are long, with waiting times for up to 3 years for an elective appointment. This rapid access pathway resulted in identifying clinically appropriate patients at the time of referral and triaging suitable cases to an in-person orthopaedic or MSK triage clinic. Appropriate patients were reviewed at these in-person clinics within 2 months of referral. If surgical intervention is a considered management option at this consultation, the patient is listed for the next Virtual UHW/MMUH Spine clinic (Fig. 1).

The Virtual (video) Spine clinic is scheduled on the first Friday of every month. Patients are listed for review at each virtual clinic, generally between 5 and 8, depending on demand. The duration of the clinic is approximately 60–90 min. The platform used for the meeting is Webex. A spinal surgeon and clinical specialist physiotherapist from MMUH link virtually (video) with the Orthopaedic team at UHW, including Orthopaedic consultants, Clinical Specialist physiotherapists, and the local pain service. Potential surgical candidates are listed for the clinic, and all relevant radiology is uploaded to the system and shared with the involved centres. Appropriate clinical documentation will be forwarded to the MMUH team before the meeting. Patients are presented by the UHW team and the MMUH Spine team gives an opinion. The three primary outcomes from the Virtual Spine clinic are (1) surgery, (2) pain intervention, or (3) continuing conservative management, e.g. physiotherapy.

**Fig. 2:**
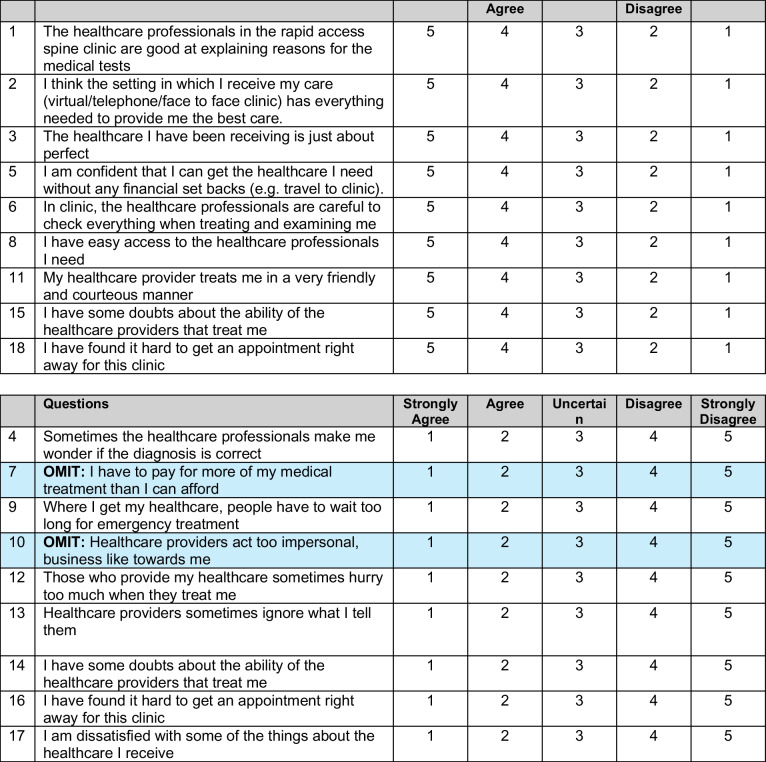
Adapted version of the PSQ-18

Retrospectively, collected data on patients discussed were analysed. Our primary aim is to measure efficiency by assessing time to initial consultation and time to virtual consultation. Secondary aims measured virtual clinic outcomes and patient satisfaction using an adapted patient-satisfaction questionnaire (PSQ-18), reflecting the unique nature of patient experience in this pathway (Fig. 2). In addition, a semistructured short interview was also carried out to assess patient satisfaction and obtain feedback on patient experience with this new novel pathway.

## Results

### Quantitative results

Forty-four patients assessed through the rapid access spine clinic were discussed at the virtual spine MDT clinic between 1 October 2021 and 2 September 2022. The median time from referral to review at an in-person rapid access physiotherapist combined orthopaedic clinic was 185 days. However, it is pertinent to note that many patients were already on a wait list before being triaged onto the novel pathway. The median time from initial consultation to virtual consultation was 36 days. The median time interval from virtual consultation to intervention was 110 days. Twenty percent of patients underwent surgery, 14% were further seen in the MMUH outpatients, 7% managed with a trial of physiotherapy, 7% required no follow-up, and 50% planned for radiologically guided spinal injections.

Table 1 exhibits patient satisfaction scores with the novel virtual spine clinic pathway. The mean (± SD) of patient satisfaction score was the highest in terms of service orientation, particularly “communication” (4.82 ± 0.39), followed by “communication” by healthcare professionals (4.72 ± 0.5). The mean (± SD) of patient satisfaction score was the lowest regarding the service accessibility and convenience subscale (3.55 ± 1.11). Two items were omitted, given that the service provision occurred in a publicly funded national university hospital.

**Table 1:** Subscale reliability of PSQ-18 and health service satisfaction scores (*n* = 44)

Subscale and Item	Mean	SD
**General satisfaction (mean = 4.17, SD = 0.85)**		
The healthcare I have been receiving is just about perfect	4.34	0.68
I am dissatisfied with some of the things about the healthcare I receive	4.00	0.96
**Technical quality (mean = 4.60, SD = 0.55)**		
I think the setting in which I receive my care (virtual/telephone/face to face clinic) has everything needed to provide me the best care	4.59	0.54
Sometimes the healthcare professionals make me wonder if the diagnosis is correct	4.41	0.54
In clinic, the healthcare professionals are careful to check everything when treating and examining me	4.75	0.53
I have some doubts about the ability of the healthcare providers that treat me	4.64	0.53
**Interpersonal manner (mean = 4.82, SD = 0.39)**		
Healthcare providers act too impersonal, business like towards me	N/A	N/A
My healthcare provider treats me in a very friendly and courteous manner	4.82	0.39
**Communication (mean = 4.72, SD = 0.50)**		
The healthcare professionals in the rapid access spine clinic are good at explaining reasons for the medical tests	4.70	0.55
Healthcare providers sometimes ignore what I tell them	4.73	0.45
**Financial aspects (mean = 4.27, SD = 0.62)**		
I am confident that I can get the healthcare I need without any financial set backs (e.g. travel to clinic)	4.27	0.62
I have to pay for more of my medical treatment than I can afford	N/A	N/A
**Time spent with healthcare professional (mean = 4.72, SD = 0.48)**		
Those who provide my healthcare sometimes hurry too much when they treat me	4.66	0.53
I have some doubts about the ability of the healthcare providers that treat me	4.77	0.42
**Accessibility and convenience (mean = 3.55, SD = 1.11)**		
I have easy access to the healthcare professionals I need	4.09	0.74
Where I get my healthcare, people have to wait too long for emergency treatment	2.91	1.16
I have found it hard to get an appointment right away for this clinic	3.18	1.21
I have found it hard to get an appointment right away for this clinic	4.00	0.96

### Qualitative results

Analysis of the semi-structured interview can be outlined into three main domains: expectations of the waiting time to index appointment, general satisfaction with the initial physiotherapy assessment visit, and preferences for virtual multidisciplinary team discussions.

#### Expectations of waiting time *to index* appointment

Patients cited quick assessment times and thorough assessments as aspects of the service they were pleased with.

- Ideally, much quicker than the traditional waiting times. I think we’re used to long waiting times in healthcare. (009)
- I think everyone should be seen as soon as possible, given the pain I experienced. Thankfully, I was given an appointment quickly enough. (014)

#### General satisfaction with the initial physiotherapy assessment visit

The vast majority of comments related to aspects of service and satisfaction with the interpersonal manner of our MSK team. Several participants expressed gratitude for the service provided.

- I was very satisfied; I couldn’t fault anyone. (022)
- The physiotherapist who had seen me was lovely. (037)

Communication is a two-way process that involves both talking and listening. Two participants identified being “heard” through effective, attentive listening as essential. Empathy, through acknowledging and understanding, was also identified as a critical component of patient satisfaction.

- They listened to me. I felt as if my pain was finally heard. (008)
- It went very well. Everyone was very friendly, and the options were laid out very clearly. (023)
- I was delighted to be listened to. Everyone was very professional and accommodating. (019)

Furthermore, a sense of psychological “relief” was expressed by participants related to receiving a diagnosis and being able to make sense of their symptoms. Patients were asked if they felt reassured after their clinical visit.

- The physiotherapist was excellent. She put my mind at ease and gave me hope that I could get back to walking without so much pain. (015)

#### Preferences for virtual multidisciplinary team discussions

When asked if patients agreed that this service reduces their clinical visits by their cases being discussed online with a spinal surgeon and if patients prefer to have their conditions discussed virtually as compared to an in-person clinical visit, the responses were almost unilaterally in support of the piloted virtual spine discussion pathway. The leading proponents include shorter times to definitive treatment and the avoidance of traffic congestion and long-distance travel into Ireland’s capital (Dublin). The latter is especially true for patients with sciatica, which can worsen with prolonged sitting.

- Yes, virtual discussions make total sense. I wouldn’t have been able to go to Dublin with the pain I was in! (011)
- I would give anything a go, as long as it’s quickest (008)
- If it speeds up the process! (035)
- I would avoid physical trips to clinics if I could. (027)
- It's perfect. I cannot imagine driving in Dublin city. (016)

## Discussion

Low back pain (LBP) is recognised as the foremost contributor to global disability. It is a common condition experienced by most people at some point in their lives. In 2020, LBP accounted for 8.1% of all-cause years lived with disability globally. This equates to approximately one out of every 13 individuals (total of 619 million) who were afflicted with LBP. Projections suggest an escalation in LBP incidences to approximately 843 million by 2050, predominantly in the African and Asian regions, attributed to demographic expansion and enhanced life expectancy [3, 4]. This poses several challenges to the orthopaedic service in Ireland.

Individuals presenting with a spectrum of lumbar region-related pathologies constitute a substantial segment of the referrals directed towards orthopaedic surgeons [5–7]. GP referrals of back pain are naturally very common. Spine centres do not have the capacity to review all referrals nationally. Most of these patients are not considered surgical candidates, contributing significantly to consultation wait times for surgery for those who need it. The majority can be assessed locally by MSK triage service and opinions by non-spine orthopaedic surgeons so that tertiary referral centres are not overwhelmed.

At a local non-spinal orthopaedic level, minimising non-surgical consultations within a surgeon’s caseload can abbreviate wait periods for surgical candidates potentially benefiting from spinal procedures and expedite redirecting non-operative cases to alternative treatment modalities [5]. Based on demographic data from European countries with established conservative spinal treatment pathways, it is estimated that there is an annual need for spinal intervention in approximately 1000 cases per million population. In Ireland, this equates to approximately 5000 cases per year [8].

From a national orthopaedic framework point of view, the National Model of Care for Trauma and Orthopaedic Surgery in Ireland identifies three major spine centres in Dublin, Cork & Galway. These centres, located at Mater Misericordiae University Hospital, Cork University Hospital, and University Hospital Galway, respectively, have seen an overwhelming volume of patients requiring outpatient assessments and surgeries. This has limited the number of spine cases performed at hospitals outside these highly specialised, high-volume centres.

Furthermore, with the evolution of sub-specialisation[9], increased surgical complexity and rising patient expectations[10], the “general” orthopaedic surgeon who does some minor spine surgery is no longer within the remit of practice for Irish orthopaedic surgeons without spinal subspecialty training. Furthermore, spine surgery is concentrated in high-volume units, and as a result, the number of orthopaedic units offering spine surgery has significantly decreased. This has led to the referral of routine and low-complexity spinal cases to regional or national centres [8].

The physiotherapy musculoskeletal (MSK) triage service, established in 2012, consists of experienced physiotherapists working closely with orthopaedic surgeons. Patients are given appointments based on how long they have been waiting for care. Approximately 85% of these patients are managed without being referred to a consultant orthopaedic clinic. Of the 15% referred for surgical evaluation, about 5% undergo spinal surgery [8]. Nonetheless, The current process for accessing care for these conditions is lengthy, with patients first consulting their primary care physician and possibly receiving analgesia, anti-inflammatories, and an exercise programme before being referred for an MRI if their condition does not improve. Depending on the results of the MRI, onward referrals are made via two main pathways: patients may be referred to a physiotherapy musculoskeletal triage clinic for non-urgent degenerative conditions or to a local non-spinal orthopaedic specialist for assessment and further workup. In cases where symptoms or neurological deficits persist, an additional wait for an appointment at a tertiary referral centre may be necessary [8].

A faster mechanism for that spinal surgical opinion is necessary when regional centres, such as UHW, are fundamentally under-resourced. There are currently over 11,700 patients waiting for Orthopaedic surgery and 76,000 waiting for an Orthopaedic outpatient appointment nationally. In addition, the Report of the National Task Force on Medical Staffing (Hanly report) from 2003 stipulated that Ireland requires a minimum of four Orthopaedic Consultants per 100,000 population to maintain a basic standard of care [2]. As of 2022, Ireland has only 2.4 orthopaedic consultants per 100,000 population, starkly contrasting to the UK and New Zealand, where the figure exceeds six orthopaedic consultants per 100,000 individuals [1]. Ideally, increasing consultant numbers is the key solution to address the lengthy outpatient waitlists. While sufficient investment in our regional orthopaedic centres is still lacking, novel pathways and virtual MDTs have become a valuable necessity to help bridge the gap.

The economic evaluation of this Rapid Access Spine pathway needs to be explored more, as it represents a significant void in evidence considering the substantial expenses related to back pain for individuals and society. Before engaging in cost-effectiveness analyses, it is crucial to have a comprehensive grasp of the health service delivery mechanisms and pathways, notably within multidisciplinary healthcare settings [11–13]. Although virtual MDTs are not resource-neutral, as the burden of monthly discussion takes clinician and specialist time at regional centres and tertiary sites, it is a low-cost alternative to appointing more surgeons in the interim.

The growing adoption of multidisciplinary care essentially took place following the recommendations of the Calman-Hine report in 1995 [14]. A concerted push for integrating multidisciplinary teams (MDTs) to guarantee the prompt and fitting delivery of care given the multiple facets of orthopaedic care. By promoting enhanced coordination and communication, MDTs have significantly improved the decision-making processes among specialists, resulting in substantial advantages for patient care, as substantiated by numerous studies [15, 16]. These teams encompass diverse healthcare professionals, each offering unique expertise to enhance patient outcome [17, 18]. This virtual spine MDT clinic includes a spinal surgeon and spinal clinical specialist physiotherapist, in addition to the non-spinal orthopaedic consultants, clinical specialist physiotherapists and pain specialists from the offsite referring hospital. Implementing this new referral pathway also stems from the forced virtualisation of MDTs by the unprecedented strain from the COVID-19 pandemic, allowing for remote specialist care delivery to a certain extent.

The quantitative and qualitative results of the patient satisfaction survey demonstrated very high satisfaction levels with the rapid access pathway/virtual spine clinic in our study. Satisfaction in healthcare is a complex entity, where individuals might appreciate some aspects of their care yet find others lacking. The parameters commonly included in standardised assessments of satisfaction within healthcare contexts cover a range of factors: the manner of personal interactions, technical excellence, accessibility and convenience, financial implications, the efficacy and outcomes of care, continuity of service, the physical environment of care facilities, and service availability [19].

PSQ-18 is comprised of 18 items with seven dimensions which measure general satisfaction (two items), technical quality (four items), interpersonal manner (two items), communication (two items), financial aspects (two items), time spent with the doctor (two items), and accessibility and convenience (four items). These items were scored on a five-point Likert scale ranging from one (strongly agree) to five (strongly disagree). Each dimension that explores tangible priorities and patient experience regarding health service satisfaction is evaluated through different related questions that identify a particular area for improvement. Some PSQ-18 items are worded so that agreement reflects satisfaction with medical care, whereas other items were worded so that agreement reflects dissatisfaction with medical care. We reversed the scores of items worded that reflected disagreement with medical care to tabulate the total satisfaction score. A higher score reflected greater satisfaction with medical care. After item scoring, items within the same subscale were averaged to create the seven subscale scores [20]. We have adapted the PSQ-18 for context as healthcare services were publicly funded by the Health Service Executive (HSE) in Ireland. Therefore, two questions concerning the transactional nature of self-funded healthcare were omitted—“I have to pay for more of my medical treatment than I can afford” and “Healthcare providers act too impersonal, business-like towards me”. However, the mean score for accessibility and convenience was lower than the other subsections as most of our data points originate from existing long-time list waiters who have been brought into the Rapid Access Spine pathway.

Integrating open-ended and closed-ended queries is advocated to garner a comprehensive insight into patient satisfaction and experiences. Slade et al. differentiate between “satisfaction” and “experience” surveys within the patient context. Satisfaction surveys, characterised by closed-ended questions, evaluate elements deemed significant by researchers and healthcare providers. Conversely, experience surveys, employing open-ended questions, position healthcare consumers, particularly those managing chronic conditions, as authorities in evaluating service quality based on their firsthand experiences [5, 21]—this study’s semi-structured interview section aimed to capture the patient experience within the Rapid Access Spine pathway.

Some limitations of this cohort study must be acknowledged. Firstly, the sample size of 44 patients may limit the generalizability of the findings to broader populations, as it may not capture the diverse experiences and outcomes of a larger patient group. Additionally, the study’s design as a cohort study may introduce selection bias, given that participants were not randomly selected, potentially affecting the representativeness of the results. The average and median waiting time, a critical measure of the pathway’s effectiveness, did not show a significant reduction. This outcome can be attributed to the fact that patients incorporated into this pathway had already experienced extended wait times before its initiation. This prior waiting period could skew the data, presenting a temporal malalignment in accurately assessing the impact of the rapid access pathway on reducing wait times. The pre-existing wait times before the introduction of the pathway may also influence patient satisfaction scores, as initial expectations may not align with the actual speed of service delivery experienced under the new system.

## Conclusion

Although still in its infancy during the cohort study period, this novel pathway efficiently serves orthopaedic units without a dedicated spinal service. Patients who have been through this pathway are generally satisfied qualitatively and quantitatively. This pathway can easily be replicated across other orthopaedic centres with minimal cost implications in the context of evolving orthopaedic service provision in Ireland.
